# Association between depression and risk of type 2 diabetes and its sociodemographic factors modifications: A prospective cohort study in southwest China

**DOI:** 10.1111/1753-0407.13453

**Published:** 2023-08-15

**Authors:** Yanli Wu, Min Chen, Tao Liu, Jie Zhou, Yiying Wang, Lisha Yu, Ji Zhang, Kunming Tian

**Affiliations:** ^1^ Guizhou Center for Disease Control and Prevention Guiyang China; ^2^ Department of Preventive Medicine, School of Public Health Zunyi Medical University Zunyi China; ^3^ Department of Geriatric Nursing, School of Nursing Zunyi Medical University Zunyi China

**Keywords:** cohort study, depression, the Patient Health Questionnaire‐9 (PHQ‐9), type 2 diabetes, 患者健康问卷‐9(PHQ‐9), 抑郁症, 2型糖尿病, 队列研究

## Abstract

**Background:**

Depression may be associated with the risk of developing type 2 diabetes. The goal of this study was to explore the association of severe of depression with the risk of type 2 diabetes in adults in Guizhou, China.

**Methods:**

A 10‐year prospective cohort study of 7158 nondiabetes adults aged 18 years or older was conducted in Guizhou, southwest China from 2010 to 2020. The Patient Health Questionnaire‐9 (PHQ‐9) was used to measure the prevalence of depression. Cox proportional hazard models were used to estimated hazard ratios (HRs) and 95% confidence intervals (95% CIs) of depression and incident type 2 diabetes. A quantile regression (QR) analytical approach were applied to evaluate the associations of PHQ‐9 score with plasma glucose values.

**Results:**

A total of 739 type 2 diabetes cases were identified during a median follow‐up of 6.59 years. The HR (95% CI) per 1‐SD increase for baseline PHQ‐9 score was 1.051 (1.021, 1.082) after multivariable adjustment. Compared with participants without depression, those with mild or more advanced depression had a higher risk of incident type 2 diabetes (HR:1.440 [95% CI, 1.095, 1.894]). Associations between depression with type 2 diabetes were suggested to be even stronger among women or participants aged ≥45 years (*p* < .05). There are significant positive associations of PHQ‐9 score with 2‐h oral glucose tolerance test blood glucose levels.

**Conclusion:**

Depression significantly increased the risk of incident type 2 diabetes, especially in women, participants aged ≥45 years, Han ethnicity, and urban residents. These findings highlighted the importance and urgency of depression health care.

## Graphical abstract

**Highlights**

- In this study, depression significantly increased the risk of incident type 2 diabetes and the risk increases as the degree of depression increases for a 5.2‐year median follow‐up.
- The results of this study suggest an age, sex, region and ethnicity difference in the relationship between depression and type 2 diabetes mellitus for Chinese populations.

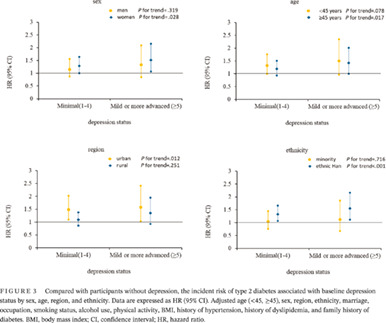

## 摘要

背景:抑郁症可能与2型糖尿病的发展风险有关。本研究旨在探索中国贵州省成年人中严重抑郁症与2型糖尿病风险的关系。

方法:在贵州省进行了一项为期10年的前瞻性队列研究, 共纳入了7,158名年龄在18岁及以上的非糖尿病患者。使用患者健康问卷‐9(PHQ‐9)测量抑郁症的患病率。采用Cox比例风险模型估计抑郁症和2型糖尿病发病的风险比值(HRs)和95%置信区间(95% CIs)。采用分位数回归(QR)分析方法评估PHQ‐9得分与血浆葡萄糖值之间的关联。

结果:在中位随访6.59年期间, 共识别出739例2型糖尿病病例。经多变量调整后, 基线PHQ‐9得分每增加1个标准差, HR(95% CI)为1.051(1.021, 1.082)。与无抑郁症状的参与者相比, 患有轻度或更严重的抑郁症的人患2型糖尿病的风险更高[HR(95% CI):1.440(1.095, 1.894)]。在女性或年龄≥45岁的参与者中, 抑郁症与2型糖尿病之间的关联更强(P<0.05)。PHQ‐9得分与口服葡萄糖耐量试验(OGTT 2h)血糖水平之间存在显著正相关关系。

结论:抑郁症显著增加了2型糖尿病的发病风险, 尤其是在女性、年龄≥45岁、汉族和城市居民中。这些发现强调了抑郁症医疗保健的重要性和紧迫性。

## 1 INTRODUCTION

Diabetes is one of the fastest‐growing diseases worldwide and poses a serious challenge to global public health, approximately 537 million adults (20–79 years) are living with diabetes in 2021 worldwide.^1^ The prevalence of type 2 diabetes in adults in China has increased from 10.4% in 2013 to 11.9% in 2018.^2^, ^3^ With the largest number of diabetes patients in the world, China bears a heavy burden of the disease.

Depression is a leading cause of disability around the world and contributes greatly to the global burden of disease. Globally, it is estimated that 5% of adults suffer from the disorder.^4^ A recent systemic analysis reported that 2.2% and of men and 3.3% of women in China suffered from major depressive disorder.^5^

Previous studies have shown that depression may be associated with the risk of developing type 2 diabetes mellitus.^6^, ^7^, ^8^, ^9^ Both major depression and diabetes are associated with hypothalamic–pituitary–adrenal axis dysfunction, which manifests as blunted diurnal cortisol rhythm.^10^ Disrupted sleep patterns are seen in people with major depression,^11^ and poor sleep quality and altered circadian rhythms are associated with insulin resistance and type 2 diabetes risk.^12^ However, most studies have been conducted in western populations, but few studies have explored the association between depression and diabetes in the Chinese population. Previous study evaluated the relationship between major depression and type 2 diabetes mellitus among Chinese people^8^ but without assessing the risk of type 2 diabetes among people with different degrees of depression. In 2019, another study investigated the association between the risk of type 2 diabetes and depression in middle‐aged and elderly Chinese,^9^ but studies on all age groups were lacking. In addition, the aforementioned studies did not conduct an in‐depth analysis on different gender, age, region, and ethnicity. Therefore, it is significant to explore the association between depression and the incidence of diabetes in the Chinese population. This study aimed to identify the influence of depression on the risk of type 2 diabetes mellitus in adults in southwest China and explored whether those associations were modified by age, sex, region, or ethnicity factors.

## 2 METHODS

### 2.1 Study population

**FIGURE 1:**
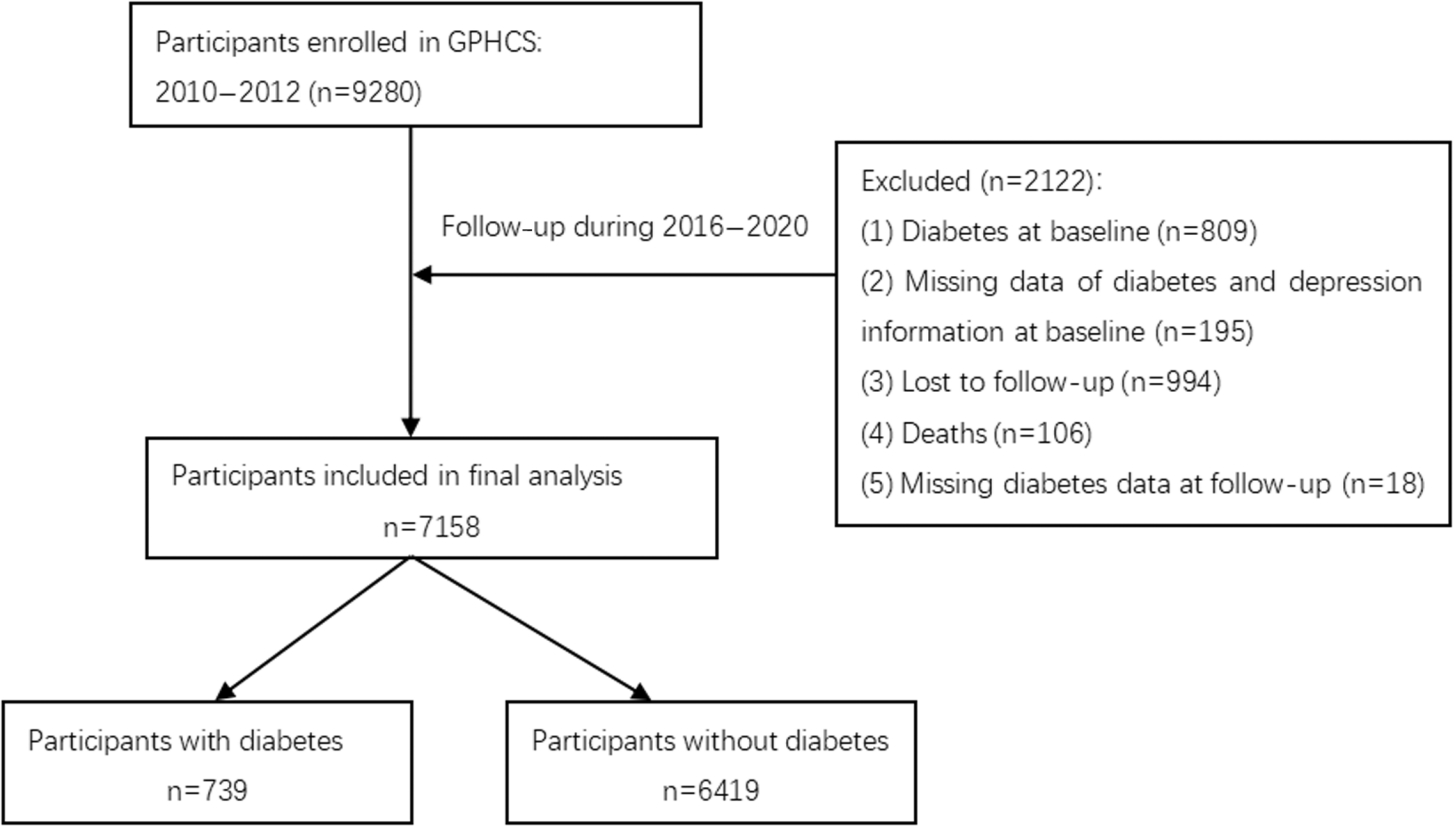
The flow chart of the study. GPHCS, Guizhou Population Health Cohort Study.

The data were obtained from the Guizhou Population Health Cohort Study, a large population database that aimed to investigate the epidemic of chronic diseases and risk factors. Briefly, a total of 9280 adult residents from 48 townships of 12 districts were recruited into this prospective cohort using the multistage proportional stratified cluster sampling method. The eligibility criteria of subjects included those who (1) were aged 18 years or above, (2) lived in the study region and had no plan to move out, (3) completed survey questionnaire and blood sampling, and (4) signed the written informed consent form. Based on the intent‐to‐treat criteria, participants were followed up from the date of entry until death, loss to follow‐up, time of a request for no further contact, or until the planned completion date in 2016–2020. During the follow‐up until 2020, information were updated on the status of major chronic diseases and vital status, with a response rate of 88%.^13^ In this study, exclusion criteria for participants were as follows: had been diagnosed with diabetes at baseline (n = 809), were not identified as having depression or diabetes owing to data missing at baseline (n = 195), were lost to follow‐up (they were not located) (n = 994), had died during the follow‐up period (n = 106), and had missing diabetes diagnosis data at follow‐up (n = 18). Finally, the remaining 7158 participants were included in the analysis (Figure 1). Among all the study subjects, the mean age was 43.63 ± 14.84 years, there were 46.90% male, 40.98% ethnic minority, 33.89% urban population, 80.61% married, and 56.83% farmers. The study was approved by the Institutional Review Board of the Guizhou Center for Disease Control and Prevention (No. S2017‐02) and written informed consent was obtained from all participants. Personal sensitive information involved in the database (such as phone number, identification number, home address, etc.) was deleted during the data analysis process in order to protect the participants' personal information and health data, and the database was protected according to Data Security Law of the People's Republic of China.

### 2.2 Assessment of depressive symptoms

The Patient Health Questionnaire‐9 (PHQ‐9) was used to screen for the presence and severity of depressive symptoms. PHQ‐9 is a self‐report questionnaire consisting of nine questions about depressive symptoms, with high internal consistency (Cronbach alpha = 0.86–0.89), and validity (sensitivity = 0.88 and specificity = 0.92).^14^, ^15^ Participants were asked to respond to each symptom by rating the best statement applied over the past 2 weeks, using a score from zero to three (ranging from“not at all”=0, “several days” = 1, “more than half the days”= 2, or“nearly every days”= 3), and the total score on the scale was 0–27; the higher score indicated the greater severity of depressive symptoms.^16^ All respondents were divided into three groups according to the PHQ‐9 scores (0, no depression; 1 to 4, minimal depressive symptoms; and ≥5, mild or more advanced symptoms as depression).^17^

### 2.3 Assessment of type 2 diabetes

Diabetes was defined according to according to the 1999 World Health Organization criteria^18^: (1) having been diagnosed with diabetes by township or community and hospitals, (2) fasting plasma glucose (FPG) ≥7.0 mmol/L (126 mg/dL), and (3) 2‐h glucose ≥11.1 mmol/L (200 mg/dL). This study classified people who meet these criteria as type 2 diabetic. The main outcome variable in this study was newly diagnosed type 2 diabetes.

### 2.4 Assessment of covariates

Information on the covariates were collected through face‐to‐face questionnaires survey and physical measurements by trained health workers. The survey included sociodemographic characteristics (age, sex, ethnicity, region, marriage status, and occupation), lifestyle habits (smoking status, alcohol use, and physical activity), history of hypertension and dyslipidemia, and family history of diabetes. Physical measurements including height, body weight, and blood pressure. Never smoker was defined as those who had never smoked. Never drinker was defined as those who had never consumed alcohol. Physical activity was defined as having moderate or vigorous physical activity at least 10 min every time for three or more times per week. Body mass index (BMI) was calculated by dividing the weight by height squared (kg/m^2^). After at least 8 h of overnight fasting, a 75 g 2 h oral glucose tolerance test was conducted for each participant. A venous blood sample was collected before and 2 h after glucose administration. Plasma glucose was detected using the hexokinase method within 4 h. After centrifugation, serum separated from the remaining blood samples was stored at −20°C to detect levels of triglycerides (TG), total cholesterol (TC), low‐density lipoprotein cholesterol (LDL‐C), and high‐density lipoprotein cholesterol (HDL‐C) (Olympus 400 Analyzer; Beckman Coulter, Brea, CA, USA). Those with hypertension had a systolic blood pressure ≥ 140 mm Hg and/or diastolic blood pressure ≥90 mm Hg, or self‐reported hypertension.^19^ Dyslipidemia was defined using either of the following criteria: (1) self‐reported doctor diagnosis of dyslipidemia or use of lipid regulating drugs; (2) high TC (TC ≥6.22 mmol/L); (3) high TG (TG ≥2.26 mmol/L); (4) low HDL‐C (HDL‐C <1.04 mmol/L); or (5) high LDL‐C (LDL‐C ≥4.14 mmol/L).^20^ Lastly, family history of diabetes was defined as those who self‐reported having a parent with diabetes.

### 2.5 Statistical analysis

The Statistical Package for the Social Sciences (version 26.0; IBM Corporation, Armonk, NY, USA) and R software (Version 4.1.2; R Foundation for Statistical Computing, Vienna, Austria) were used to perform statistical analyses. Data were described as means and SDs for continuous variables and as frequencies and percentages for categorical variables. Baseline characteristics were compared via the analysis of variance or the chi‐square test. Person‐years (PYs) were used as the time variable. PYs were calculated from the baseline survey to the onset of diabetes or the end of follow‐up, and the incidence density of different depression status group was calculated. A Cox proportional hazards regression model was used to evaluate the associations of depression with type 2 diabetes mellitus.

A quantile regression (QR) analytical approach was applied to evaluate the associations of PHQ‐9 score with plasma glucose levels at a set of quantile levels ranging from 0.1 to 0.9. QR can be used to compare the entire distribution of a continuous response or a specific quantile of the response between groups. The advantage is that it allows for understanding relationships between variables outside of the conditional mean of the response; it is useful for understanding an outcome at its various quantiles and comparing groups or levels of an exposure on those quantiles.^21^ The distinguishing feature of the QR model is that the regression coefficients of depression score may differ across the quantile levels of plasma glucose.

Two sensitivity analyses were conducted to assess the robustness of the results: (1) excluding participants who were diagnosed within 2 years, and (2) adjusting for the BMI as a categorical variable (BMI < 18.5 kg/m^2^,18.5 ≤ BMI < 24 kg/m^2^, BMI≥24 kg/m^2^).

The Schoenfeld residuals were used to test the assumption of hazard proportionality in Cox regression models and no evidence of nonproportionality was found (*p* ≥ .05). All analyses were two tailed and *p* < .05 was considered to indicate statistical significance.

## 3 RESULTS

### 3.1 Baseline characteristics of participants

During a median follow‐up of 6.59 years, 7158 participants at baseline were included in the analysis. The baseline characteristics of participants are presented in Table 1. Of all subjects, the average age was 43.63 ± 14.84 years old and more than half were women. A total of 462 (6.45%) participants presented with depression (PHQ‐9 score ≥5), with an average score of 7.30 ± 2.96, and nearly one fifth (19.15%) had minimal depression with the PHQ‐9 score between 1 and 4. Compared with participants without depression (PHQ‐9 score = 0), depressive ones more likely to be older, female, urban, married, nonfarmers, and nonethnic minority. They also had a higher proportion of alcohol use and a higher prevalence of hypertension, dyslipidemia, or family history of diabetes.

**TABLE 1:** General characteristics of the study population by the depression status at baseline.

Characteristics	Total	Depression status			*p* value
		No (0)	Minimal (1–4)	Mild or more advanced (≥5)	
N	7158	5325	1371	462	
PHQ‐9 score	0.88 ± 2.07	0	2.11 ± 1.05	7.30 ± 2.96	<.001
Age at baseline, years	43.63 ± 14.84	42.8 ± 14.76	46.07 ± 15	45.96 ± 14.23	<.001
<45	3967 (55.42)	3070 (57.65)	672 (49.02)	225 (48.70)	<.001
≥45	3191 (44.58)	2255 (42.35)	699 (50.98)	237 (51.30)	
Men, %	3357 (46.90)	2586 (48.56)	595 (43.40)	176 (38.10)	<.001
Ethnic minority, %	2933 (40.98)	2272 (42.67)	497 (36.25)	164 (35.50)	<.001
Urban, %	2426 (33.89)	1606 (30.16)	603 (43.98)	217 (46.97)	<.001
Married, %	5770 (80.61)	4256 (79.92)	1141 (83.22)	373 (80.74)	.022
Farmer, %	4068 (56.83)	3104 (58.29)	739 (53.90)	225 (48.70)	<.001
Physical activity, %	430 (6.01)	325 (6.10)	80 (5.84)	25 (5.41)	.799
Never smoker, %	5124 (71.58)	3789 (71.15)	984 (71.77)	351 (75.97)	.087
Never drinker, %	4897 (68.41)	3693 (69.35)	888 (64.77)	316 (68.4)	.005
Body mass index, kg/m^2^	22.77 ± 3.29	22.78 ± 3.29	22.81 ± 3.36	22.54 ± 3.1	.285
History of hypertension, %	1721 (24.04)	1230 (23.10)	376 (27.43)	115 (24.89)	.003
History of dyslipidemia, %	4833 (67.52)	3561 (66.87)	943 (68.78)	329 (71.21)	.016
Family history of diabetes, %	96 (1.34)	62 (1.16)	22 (1.60)	12 (2.60)	.024

Abbreviation: PHQ‐9, Patient Health Questionnaire‐9.

### 3.2 Associations of depression with incident type 2 diabetes

In this study, 739 diabetes occurred during the 10‐year follow‐up period. The incidence density of type 2 diabetes was 14.54 per 1000 PYs. Over the past decade, the incidence rates of type 2 diabetes among participants with depression were significantly higher than for those without depression. The incident density was highest in the depression group (18.05 per 1000 PYs), followed by minimal and no depression groups. The unadjusted (Table 2, Model 1) and age‐ and sex‐adjusted (Table 2, Model 2) Cox model showed that the PHQ‐9 score was associated with an increased risk of incident type 2 diabetes. In the fully adjusted models (Table 2, Model 3), the adjusted hazard ratios (HRs) were 1.051 (95% confidence interval [CI]: 1.021–1.082) with per SD increase of PHQ‐9 score. The risk of type 2 diabetes increases as the degree of depression increases. The risk for the group with minimal depression group (PHQ‐9 score:1–4) (HR = 1.259 [95% CI: 1.054–1.506]) and depression group (PHQ‐9 score:≥5) (HR = 1.415 [95% CI: 1.080–1.853]) were stronger than no depression. Compared with the no depression group, after adjustment for sex and age, the group with minimal depression and depression had the higher HR for type 2 diabetes, after further adjusting for ethnicity, region, marriage status, occupation, smoking status, alcohol use, physical activity, history of hypertension and dyslipidemia, and family history of diabetes, participants with minimal depression had 1.235 times (HR = 1.235 [95% CI: 1.030–1.480]) risk of type 2 diabetes and with depression had 1.440 times (HR = 1.440 [95% CI: 1.095–1.894]) (Table 2, Model 3).

**TABLE 2:** The incident risk of type 2 diabetes associated with baseline depression status.

Level of symptom severity PHQ‐9 score	Cases, n	Incident density/1000 PYs	HR (95% CI)					
			Model 1	*p* value	Model 2	*p* value	Model 3	*p* value
PHQ‐9 score (per SD increase)	739	14.54	1.051 (1.021, 1.081)	.001	1.047 (1.017, 1.077)	.002	1.051 (1.021, 1.082)	.001
No (0)	523	13.80	1.000		1.000		1.000	
Minimal (1–4)	157	16.26	1.259 (1.054, 1.506)	.011	1.219 (1.019, 1.458)	.030	1.235 (1.030, 1.480)	.023
Mild or more advanced (≥5)	59	18.05	1.415 (1.080, 1.853)	.012	1.370 (1.046, 1.796)	.022	1.440 (1.095, 1.894)	.009
*p* for trend				.004		.014		.006

*Note*: Model 1: unadjusted. Model 2: adjusted age (<45, ≥45), sex. Model 3: model 2 plus region, ethnicity, marriage, occupation, smoking status, alcohol use, physical activity, body mass index, history of hypertension, history of dyslipidemia, and family history of diabetes.

Abbreviations: CI, confidence interval; HR, hazard ratio; PHQ‐9, Patient Health Questionnaire‐9.

**FIGURE 2:**
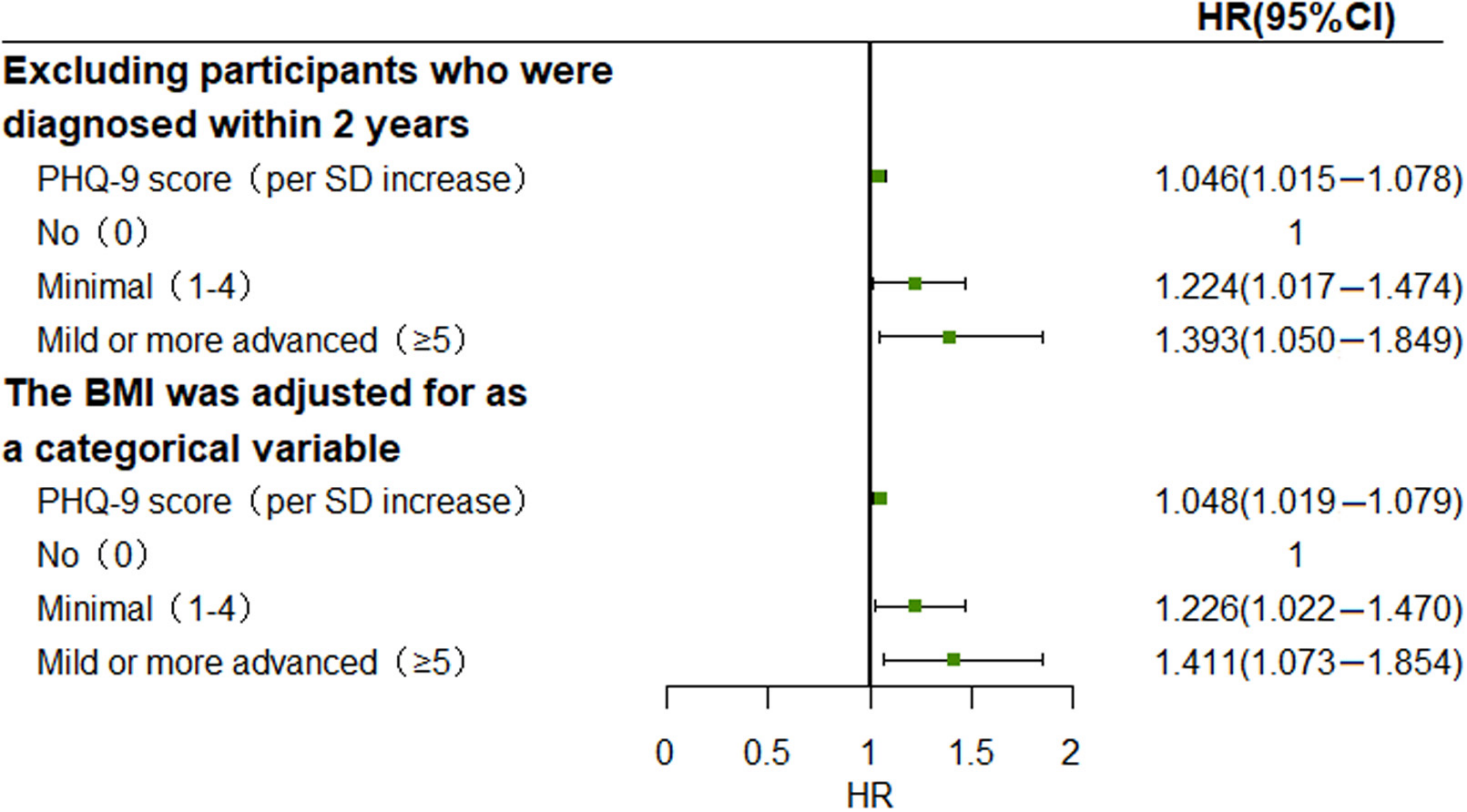
Sensitivity analysis. Two sensitivity analyses were conducted to assess the robustness of the results. Participants who were diagnosed within 2 years were excluded, and the BMI was adjusted for as a categorical variable (BMI < 18.5 kg/m^2^, 18.5 ≤ BMI < 24 kg/m^2^, BMI≥24 kg/m^2^). HR (95% CI) refers to the risk of type 2 diabetes per 1‐SD increase for PHQ‐9 score, or compared with participants without depression, the risk of type 2 diabetes in those with minimal and any mild or more advanced depression. Adjusted age (<45, ≥45), sex, region, ethnicity, marriage, occupation, smoking status, alcohol use, physical activity, BMI (<18.5 kg/m^2^ or ≥24 kg/m^2^, ≥18.5 and <24 kg/m^2^), history of hypertension, history of dyslipidemia, and family history of diabetes. BMI, body mass index; CI, confidence interval; HR, hazard ratio; PHQ‐9, Patient Health Questionnaire‐9.

Two sensitivity analysis were conducted by excluding participants who were diagnosed within 2 years and adjusting for BMI as a categorical variable, the results of which did not differ substantially from those of the primary analysis (Figure 2).

### 3.3 Subgroup analysis

**FIGURE 3:**
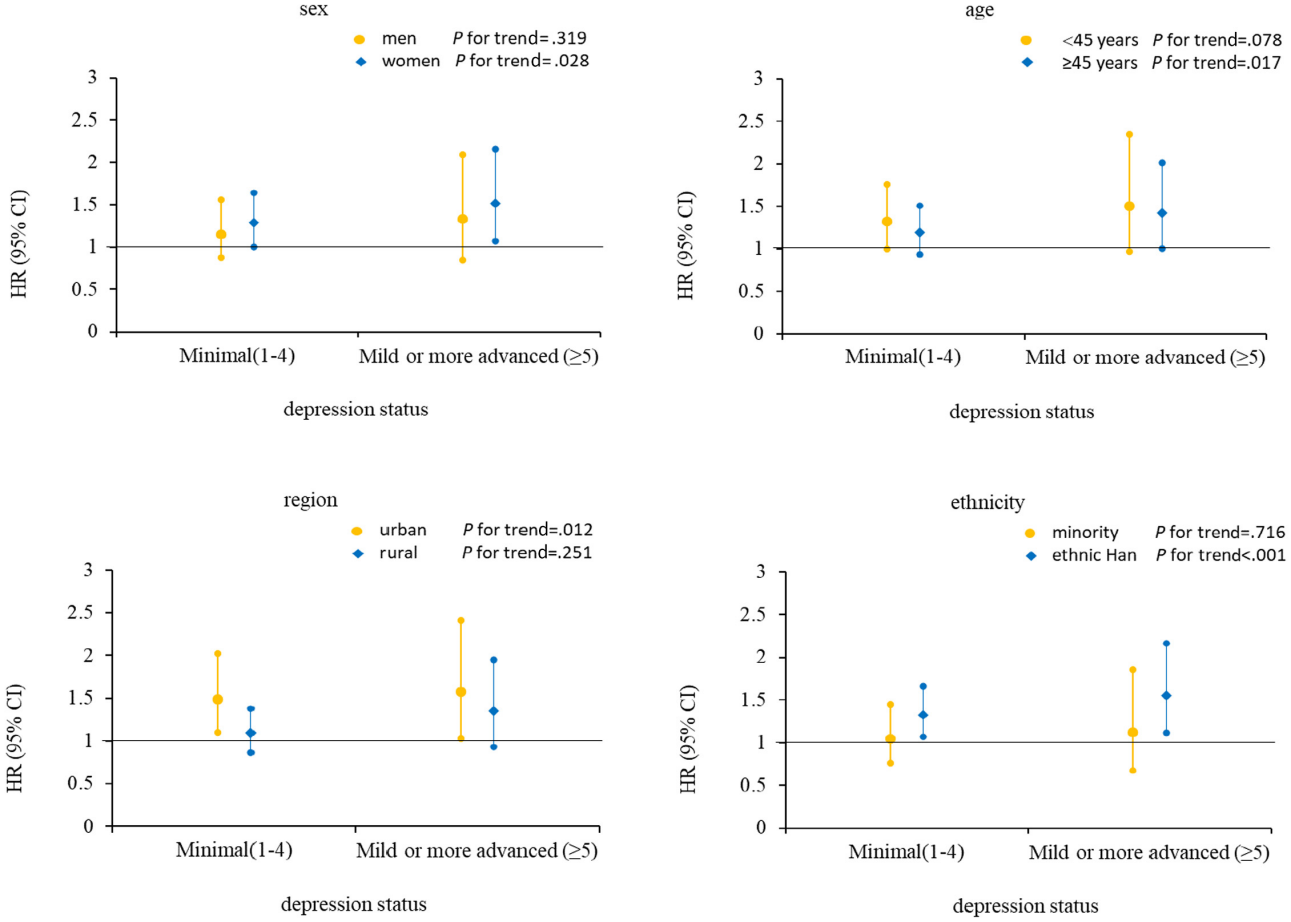
Compared with participants without depression, the incident risk of type 2 diabetes associated with baseline depression status by sex, age, region, and ethnicity. Data are expressed as HR (95% CI). Adjusted age (<45, ≥45), sex, region, ethnicity, marriage, occupation, smoking status, alcohol use, physical activity, BMI, history of hypertension, history of dyslipidemia, and family history of diabetes. BMI, body mass index; CI, confidence interval; HR, hazard ratio.

This study also explored the potential effect modification of baseline age, sex, ethnicity, and region on the associations of depression with incident type 2 diabetes, and the results of the subgroup analyses are presented in Figure 3. Compared with the no depression group, participants with minimal depression and depression in women, participants aged ≥45 years, Han ethnicity, or urban residents had higher risk of type 2 diabetes than men, those aged <45 years, minority group members, or rural residents.

### 3.4 Associations of PHQ‐9 score with plasma glucose levels

**FIGURE 4:**
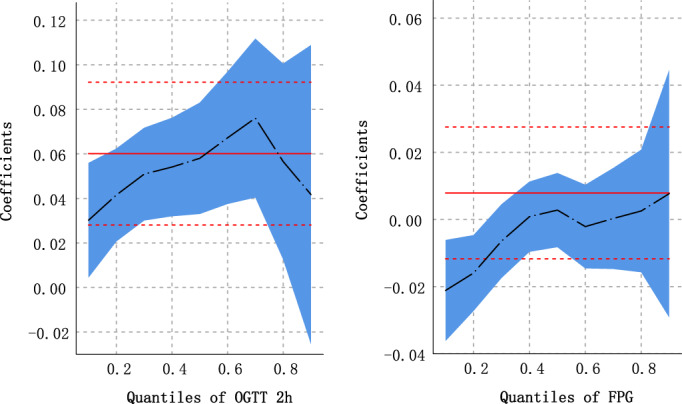
Coefficients (β) for the associations of plasma glucose levels (OGTT 2 h and FPG) with PHQ‐9 across the quantile levels of plasma glucose levels. The coefficients indicate the change in plasma glucose levels (mmol/L) with one unit (1 score) increased of PHQ‐9 score. Adjusted age (<45, ≥45), sex, region, ethnicity, marriage, occupation, smoking status, alcohol use, physical activity, BMI, history of hypertension, history of dyslipidemia, and family history of diabetes. The black line represents parameter estimates at the different regression quantiles; the blue area represents confidence intervals of the parameter estimates; the red line represents parameter estimates for the ordinary linear regression with the same predictors; the red dots confidence interval bounds for the ordinary linear regression with the same predictors. BMI, body mass index; FBG, fasting plasma glucose; OGTT, oral glucose tolerance test; PHQ‐9, Patient Health Questionnaire‐9.

The coefficient estimates and 95% CI for the associations of plasma glucose levels with PHQ‐9 score across the quantile levels of plasma glucose are given in Table 3 and Figure 4. According to the ordinary least‐squares estimations, a significant positive association was observed between PHQ‐9 score at baseline and oral glucose tolerance test (OGTT) 2‐h plasma glucose levels during the follow‐up survey (β:0.059 [95% CI: 0.028–0.091]), that is, OGTT 2‐h plasma glucose increased by 0.059 mmol/L with each 1‐point increase in PHQ‐9 score. The results of the QR show that within the range of 0.1 to 0.7 quantile levels of OGTT 2 h, the higher the quantile level, the greater the effect of PHQ‐9 score on OGTT ‐ h plasma glucose level. OGTT 2‐h plasma glucose increased by 0.031 (95% CI: 0.005–0.058) mmol/L with each 1‐point increase in PHQ‐9 score at the quantile of 0.1, and OGTT 2‐h plasma glucose increased by 0.072 (95% CI: 0.037–0.107) mmol/L at 0.7. When the quantile level was higher, the effect of PHQ‐9 score on OGTT 2‐h plasma glucose value was weakened, 0.054 (95% CI: 0.009–0.098) at 0.8, and no statistically significant at 0.9 (β: 0.051, 95% CI: −0.015 to 0.117). A significant effect was not observed about PHQ‐9 score on increasing FPG.

**TABLE 3:** Associations of plasma glucose levels with PHQ‐9 score at the mean and selected quantile levels of plasma glucose.

Quantile levels of plasma glucose	2hPG		FPG	
	Mean value or corresponding value at the quantile levels (mmol/L)	Coefficients^a^ (95% CI)	Mean value or corresponding value at the quantile levels (mmol/L)	Coefficients^a^ (95% CI)
OLS	6.784	0.059 (0.028 to 0.091)	5.510	0.008 (−0.011 to 0.026)
QR				
0.1	4.003	0.031 (0.005 to 0.058)	4.136	−0.021 (−0.036 to −0.006)
0.2	4.380	0.040 (0.019 to 0.062)	4.366	−0.016 (−0.028 to −0.005)
0.3	4.801	0.047 (0.026 to 0.068)	4.582	−0.007 (−0.018 to 0.004)
0.4	5.136	0.050 (0.027 to 0.072)	4.710	−0.001 (−0.011 to 0.010)
0.5	5.461	0.058 (0.034 to 0.083)	4.915	0.001 (−0.010 to 0.012)
0.6	5.818	0.065 (0.035 to 0.094)	4.988	−0.002 (−0.015 to 0.010)
0.7	6.519	0.072 (0.037 to 0.107)	5.196	−0.001 (−0.016 to 0.013)
0.8	6.611	0.054 (0.009 to 0.098)	5.355	−0.003 (−0.021 to 0.015)
0.9	7.320	0.051 (−0.015 to 0.117)	5.626	0.007 (−0.026 to 0.041)

*Note*: 132 participants who took hypoglycemic drugs were excluded.

Abbreviations: 2hPG, 2‐h postload plasma glucose; CI, confidence interval; FPG, fasting plasma glucose; OLS, ordinary least squares; QR, quantile regression.

^a^ Refers to the change in plasma glucose levels (mmol/L) with one unit (1 score) increased of PHQ‐9 score. Adjusted age (<45, ≥45), sex, region, ethnicity, marriage, occupation, smoking status, alcohol use, physical activity, body mass index, history of hypertension, history of dyslipidemia, and, family history of diabetes.

## 4 DISCUSSION

Based on a prospective cohort study in southwest China, depression symptom severity was positively associated with increased risk for type 2 diabetes, especially among women, participants aged ≥45 years, Han ethnicity, and urban residents. At the same time, this study found that different quantile levels of OGTT 2‐h plasma glucose had different effects of PHQ‐9 score on OGTT 2‐h plasma glucose, Within a certain range, the higher the quantile level, the greater the effect of PHQ‐9 score on OGTT 2‐h plasma glucose level. These findings indicated that improving depression may help to prevent and control developing type 2 diabetes.

Previous studies have suggested that depression is associated with an increased risk of diabetes.^8^, ^9^, ^22^ Meng et al performed a median follow‐up of 7.2 years survey of 461 213 participants. They found that a major depressive symptoms episode, defined as responding positively to the screening question and having three or more of seven diagnostic symptoms for the modified Chinese version of Composite International Diagnostic Interview Short‐Form was associated with a 1.31‐fold greater hazard of type 2 diabetes.^8^ In another cohort of the China Health and Retirement Longitudinal study among 47 671 middle‐aged and older adults, Luo et al reported that participants with depressive symptoms had a 1.33‐time higher risk of incident type 2 diabetes.^9^ As expected, the depressive symptoms were associated with a higher risk of type 2 diabetes in this study. Minimal depression was associated with 1.235‐fold risk of type 2 diabetes, and risk of depression was 1.440‐fold. These findings further support the current evidence that depression is a risk factor for type 2 diabetes. A few studies have found that the association between depression and diabetes could be bidirectional,^23^ and type 2 diabetes was also associated with an increased risk of depression. Thus, to decrease the possibility of reverse causation, type 2 diabetes cases diagnosed within the previous 2 years were excluded, and the results remained unchanged after conducting the sensitivity analysis. A meta‐analysis to examine the bidirectional prospective relationships between depression and type 2 diabetes also showed that the relative risk for incident diabetes associated with baseline depression was 1.60 (1.37–1.88). Depression is associated with a 60% increased risk of type 2 diabetes, type 2 diabetes is associated with only modest increased risk of depression.^24^

There are several potential mechanisms for the association of depression with risk of incident type 2 diabetes. First, depression may increase the risk of developing type 2 diabetes through physiological changes, for example, activation of the hypothalamic–pituitary–adrenal axis and the immune system, paving the way for development of type 2 diabetes.^10^, ^25^ Second, depression and type 2 diabetes under unified used similar environmental factors, including childhood adversity experiences and socioeconomic deprivation.^26^ Third, patients with depression often had unhealthy lifestyles, such as smoking, little physical activity, and excessive calorie and alcohol intake.^27^, ^28^ These unhealthy lifestyle habits often increased the risk of diabetes.^29^, ^30^ Additionally, the usage of antidepressants by people with elevated depressive symptoms might affect the incidence of type 2 diabetes. Long‐term use of antidepressants in at least moderate daily doses was related to an increased risk of type 2 diabetes.^31^ Nevertheless, this study has not been able to gain information on antidepressant use and could not examine this hypothesis during the study. But in China's Kadoorie Biobank, only 15% of participants with depression sought help from a doctor,^32^ so the effect of antidepressants may be small. Finally, some researchers suggested that this known increased risk might result in part from an augmented awareness of both depression and type 2 diabetes, leading to more diagnoses of depression in this population.^33^ Besides, this study found that the PHQ‐9 score had only a positive effect on OGTT 2‐h glucose values but not significantly on FPG, which requires more studies to confirm and the biological mechanisms deserve further investigation.

In the stratified analysis by sex or age found that the effects of depression on incident type 2 diabetes were higher in women or participants aged ≥45 years, which was consistent with the previous studies.^8^, ^9^ This study also observed a greater impact of depression on diabetes in the urban population than rural. The possible reason is that there is a lower risk of depression among rural dwellers,^34^ and the features of cities (such as artificial light, neighborhood crime) could increase depression risk among urban older adults via disrupted sleep.^35^ The prevalence of depression is also higher in urban than rural dwellers in this study. Most important, in this study, ethnicity modified associations between depression and incident type 2 diabetes. Participants with depression has a higher risk of type 2 diabetes in people of Han than minority ethnicity. Several studies have shown ethnic disparities in depression^36^, ^37^ ethnic differences in depression may affect ethnic differences in incident type 2 diabetes, although the biological mechanism was not yet clear to us. Even so, these findings provide new evidence that there may be an ethnic difference in the association of depression with incident type 2 diabetes in the Chinese population.

The strengths of this study included the well‐characterized prospective design and the longer follow‐up period with a relatively low loss to follow‐up rate. To our knowledge, so far, no other study has been carried out to evaluate the associations of PHQ‐9 score with plasma glucose levels by applying the QR model in China. Nevertheless, several limitations should also be acknowledged. First, we only measured baseline depression status using PHQ‐9 and did not measure during the follow‐up. Also, this study did not have information about clinical diagnoses of depression, which might lead to a misclassification of the depression status. Second, some variables, such as the use of antidepressants, were not considered due to the data restrictions. Third, even though current analyses adjusted for major potential confounding factors, residual confounding resulting from dietary factors was still possible. In addition, data are inevitably missing in this large‐scale longitudinal representative study, and this study was conducted only in Guizhou Province, so the interpretation of the results needs to be cautious.

In conclusion, depression significantly increased the risk of incident type 2 diabetes, especially in women, participants aged ≥45 years, Han ethnicity, and urban residents. In the future planning of this cohort study, the association of genotypes with type 2 diabetes in populations with different demographic sociological characteristics will be studied, which will further confirm the findings. However, this results contribute new evidence on the association between depression and the risk of developing type 2 diabetes in adults in southwest China and highlight the need for focused attention on depression management and diabetes prevention, especially for the middle‐aged and elderly people, women, Han ethnicity, and urban residents.

## AUTHOR CONTRIBUTIONS

Tao Liu and Kunming Tian conceived the study. Yanli Wu performed the statistical analysis and drafted the manuscript. Min Chen performed the statistical analysis. Tao Liu, Jie Zhou, Yiying Wang, Lisha Yu, Ji Zhang, and Kunming Tian critically revised the manuscript. All authors read and approved the final version of the manuscript.

## FUNDING INFORMATION

This work was supported by Guizhou Province Science and Technology Support Program [Grant number: Qiankehe (2018)2819] and Provincial Key Construction Discipline Project by Guizhou Health Commission.

## CONFLICT OF INTEREST STATEMENT

The authors have no conflicting interests relevant to this article to disclose.

## Data Availability

Application for data sets generated during and/or analyzed during the current study may be considered by the corresponding author on reasonable request.
